# Liver Fat Content, Evaluated through Semi-Quantitative Ultrasound Measurement, Is Associated with Impaired Glucose Profiles: A Community-Based Study in Chinese

**DOI:** 10.1371/journal.pone.0065210

**Published:** 2013-07-02

**Authors:** Xiaoming Li, Mingfeng Xia, Hui Ma, Yu Hu, Hongmei Yan, Wanyuan He, Huandong Lin, Naiqing Zhao, Jian Gao, Xin Gao

**Affiliations:** 1 Department of Endocrinology and Metabolism, Zhongshan Hospital, Fudan University, Shanghai, China; 2 Department of Ultrasonography, Zhongshan Hospital, Fudan University, Shanghai, China; 3 Department of Biostatistics, College of Public Health, Fudan University, Shanghai, China; 4 Department of Evidence Base Medicine Center, Zhongshan Hospital, Fudan University, Shanghai, China; University of Verona, Ospedale Civile Maggiore, Italy

## Abstract

Nonalcoholic fatty liver disease (NAFLD) is closely associated with type 2 diabetes mellitus. We investigated whether the deposition of fat in the liver is associated with glycemic abnormalities and evaluated the contribution of the liver fat content (LFC) to the impaired glucose regulation. We conducted a community-based study among 2836 residents (1018 males and 1818 females) without prior known diabetes mellitus from the Changfeng Study who were at least 45 years old. A standard interview, anthropometrics and laboratory parameters were performed for each participant. The standardised ultrasound hepatic-renal echo-intensity and hepatic echo-intensity attenuation rate were used to assess the LFC. The cohort was stratified according to the quintiles for LFC. Two-hour glucose and fasting blood glucose increased across the LFC quintiles after adjustment for age and gender. LFC increased continuously among glucose categories after adjustment for age and gender (NGT: 7.7±0.3%, IFG: 10.0±0.8%, IGT: 11.8±0.5%, IFG+IGT: 11.7±0.9%, new- DM: 12.4±0.6%, *P*<0.001). By logistic regression analysis, 1% LFC increment independently predicted prediabetes and diabetes (OR 1.032, 1.019–1.045, *P*<0.001; 1.021, 1.005–1.037, *P* = 0.012, respectively) after adjustment for all potential confounders. Furthermore, participants with LFC higher than 10% had higher odds ratios of impaired glucose regulation as compared with those with LFC below 10% in fully adjusted logistic models. These results suggest that the LFC is strongly associated with impaired glucose regulation in the Chinese population, and that an even slightly elevated LFC is associated with increased glucose dysregulation.

## Introduction

The prevalence of nonalcoholic fatty liver disease (NAFLD) is approximately 10–30% in the general population in various countries and is considered to be increasing [1], [2]. NAFLD is frequently associated with visceral obesity, dyslipidaemia, insulin resistance and type 2 diabetes mellitus (T2DM) and may represent another component of the metabolic syndrome (Mets) [3], [4], [5], [6]. Previous studies have shown that people with NAFLD are more than twice likely to have T2DM [7], and even moderate elevation in alanine aminotransferase (ALT) levels, a poor surrogate of fatty liver, was found to be associated with high-normal glucose levels [8]. Moreover, prospective studies suggest that people with NAFLD are more likely to have T2DM at all time points of follow-up [9], [10], [11], [12], [13]. Therefore, the available evidence suggests that the deposition of fat in the liver might play a role in the development of T2DM. However, in previous studies, the diagnosis of NAFLD was commonly based on ultrasonographic findings, which could not quantify LFC or liver enzymes from laboratory tests, which have low specificity. Thus, the relationship between LFC and impaired glucose regulation is currently uncertain. Moreover, the extent to which the liver fat content (LFC) may reflect a defect in impaired glucose regulation is unclear. Clarification of these aspects may be of clinical importance with regard to planning early preventive strategies and identifying therapeutic targets.

Therefore, in the present study we investigated whether the deposition of fat measured by semi-quantitative ultrasonography in the liver is associated with glycemic abnormalities in people without prior known T2DM. In addition, we further evaluated the contribution of the LFC to the adverse glucose regulation.

## Materials and Methods

### 2.1. Study design

The subjects were participants in the Changfeng Study, a community-based study of chronic diseases among the middle-aged and elderly, that has been described elsewhere [14]. From May 2010 to March 2012, 4054 consecutive participants were initially screened. In this study, a total of 1218 of the subjects were not included due to prior known diabetes (n = 468), incomplete data (n = 218), alcohol abuse (defined as alcoholic intake ≥20 g/day for men and ≥10 g/day for women, n = 276), viral B hepatitis (n = 164) or other disease (n = 92), such as hepatic dysfunction (defined as >1.5-fold elevation of alanine aminotransferase [ALT], aspartate aminotransferase [AST], or direct bilirubin) or renal dysfunction (defined as serum creatinine >115 mmol/L). Finally, 2836 subjects (1018 males and 1818 females) were included in the analysis.

**Figure 1:**
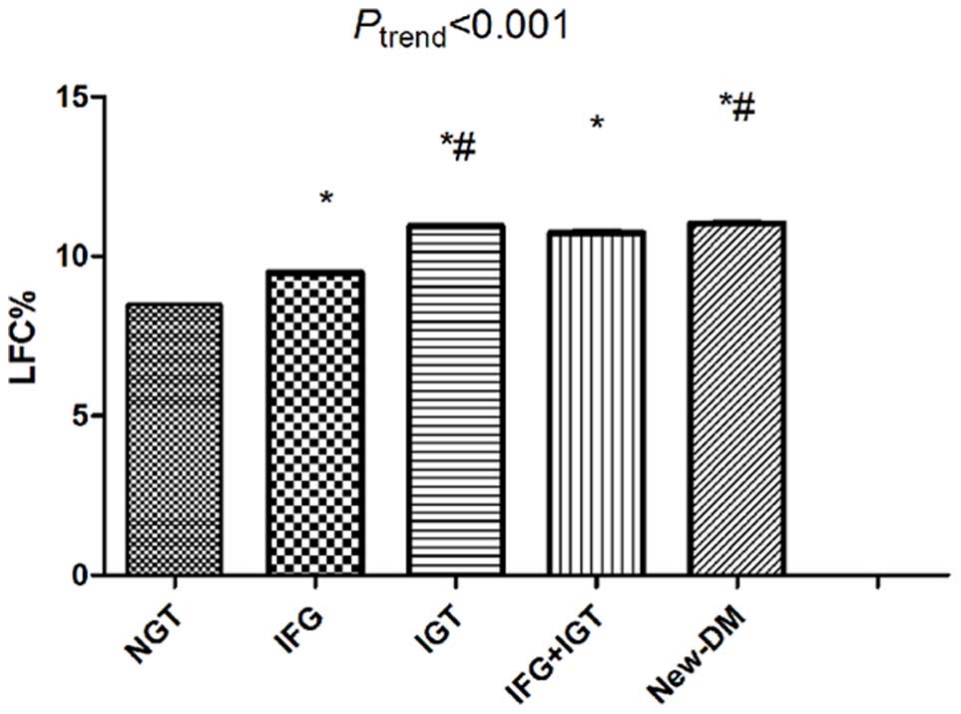
The liver fat content increases across the glucose profiles after adjustment for age and gender. *: vs. NGT; #: vs. IFG; LFC: liver fat content; NGT: normal glucose tolerance; IFG: impaired fasting glucose; IGT: impaired glucose tolerance; DM: diabetes mellitus.

This study was approved by the Research Ethics Committee of the Shanghai Health Bureau, and written informed consent was obtained from all participants. Physical examinations, laboratory assessments and ultrasound liver scans were performed on each study subject at the Changfeng Community Hospital.

### 2.2. Data collection

#### 2.2.1. Interview

Trained nurses interviewed all participants and obtained their medical history and health-related behaviours using a standardised questionnaire. Current use of medications was obtained from all participants by questionnaire. We calculated an average daily intake of alcohol from a series of questions.

#### 2.2.2. Physical examination

Weight and height were measured while the participant was clothed in a light gown. The waist circumference was measured midway between the lowest rib margin and the iliac crest in a standing position. Body mass index (BMI) was calculated as weight divided by height squared (kg/m^2^). Waist-to-hip ratio (WHR) was calculated from these values. Resting blood pressure was measured three times by an electronic blood pressure monitor (OMRON Model HEM-752 FUZZY’Omron Co., Dalian, China), and the mean value was used for the analysis.

#### 2.2.3. Laboratory assessments

Blood samples were obtained after a fasting period of at least 10 h. Total cholesterol (TC), high-density lipoprotein cholesterol (HDL-c), triglycerides (TG), uric acid (UA) and liver enzymes were measured by a model 7600 automated bio-analyser (Hitachi, Tokyo, Japan). The level of low-density lipoprotein cholesterol (LDL-c) was calculated by the Friedewald equation. The fasting blood glucose (FBG) and postload plasma glucose (PPG) of 2-h glucose levels following a 75-g oral glucose challenge for non-diabetics were measured using the glucose oxidase method.

#### 2.2.4. Ultrasonographic examination

Hepatic ultrasonography scanning was performed on all participants by an experienced radiologist who was blinded to the participants’ details using a GE Logiq P5 scanner (GE Healthcare, Milwaukee, USA) with a 4-MHz probe. The ultrasound hepatic/renal echo-intensity ratio (H/R) and ultrasound hepatic echo-intensity attenuation rate (HA) were obtained from ordinary ultrasound images using NIH-image software (Image J 1.41o, National Institutes of Health, USA) in a computer program. Both parameters were standardised using a tissue-mimicking phantom before analysis. And we measured LFC according to the method described in detail elsewhere [15]. Repeated measurements in the same subjects (performed in 102 subjects randomly selected from the 2836 participants) gave an intraclass correlation coefficient of 95%.

### 2.3. Definitions

Hypertension was defined as published in the Seventh Report of the Joint National Committee on Prevention, Detection, Evaluation, and Treatment of High Blood Pressure [16]. Glucose tolerance was evaluated based on OGTT as follows by WHO 1999 criteria [17]: normal glucose tolerance (NGT) (FBG <5.6 mmol/l and PPG <7.8 mmol/l); isolated impaired fasting glucose (i-IFG) (FBG 5.6–6.9 mmol/l and PPG <7.8 mmol/l); isolated impaired glucose tolerance (i-IGT) (fasting glucose <5.6 mmol/l and 2 h glucose 7.8–11.0 mmol/l); and IFG+IGT(FBG 5.6–6.9 mmol/l and PPG 7.8–11.0 mmol/l) [18]. Mets was defined according to the joint interim statement of the International Diabetes Federation Task Force on Epidemiology and Prevention [19].

### 2.4. Statistical analysis

Data were expressed as the means ± SD, frequencies or medians with 25th and 75th percentiles. Skewed variables were logarithmically transformed to improve normality prior to analysis. To evaluate a potential relationship between each parameter and LFC, subjects were stratified according to the LFC quintiles. The ranges of LFC in the quintiles were 0∼5%, 5∼10%, 10∼15%, 15∼20% and 20∼48.02%. An analysis of covariance and logistic regression, with adjustments for age and gender, was conducted to compare means and proportions, respectively, across the LFC quintiles. ANOVA was used to test for differences of the LFC across the different glucose profiles. Furthermore, the odds ratio (OR) and 95% confidence intervals (CIs) for the unadjusted and adjusted LFC risk factors for prediabetes and diabetes were then calculated via the logistic regression models. Multiple linear regressions were also used to evaluate the role of LFC in determining FBG and PPG during the OGTT. The independent variables entered were age, gender, ALT, AST, UA, BMI, WHR, HDL, TG, systolic blood pressure (SBP), diastolic blood pressure (DBP) and LFC. We also implemented three logistic regression models to assess the relationship between LFC categories and impaired glucose regulation. In model 1, no covariates were adjusted; in model 2, age and gender were adjusted; in model 3, age, gender, ALT, AST, UA, BMI, WHR, HDL, TG, SBP and DBP were adjusted. For all analyses, *p* value of <0.05 was considered statistically significant. All analyses were performed using SPSS 17.0 for Windows (SPSS Inc., Chicago, IL).

## Results

### Characteristics of the Subject

The mean age of the study subjects was 62.58±9.67 years, the mean BMI was 24.11±3.30 kg/m^2^. I-IFG, i-IGT, IFG+IGT and new diabetes mellitus were identified in 9.20%, 14.60%, 6.88% and 12.87% of the subjects, respectively. And hypertension, dyslipidaemia and Mets were identified in 62.30%, 47.95% and 36.92% of the participants, respectively. The prevalences of people taking anti-hypertension, anti-platelet and lipid-lowering medications were 34.30%, 10.16% and 7.07%, respectively (data not shown).

### Anthropometric and Metabolic Phenotypes According to the LFC (Table 1)

Table 1 lists the demographic characteristics of the participants stratified by the LFC quintiles. Across the quintiles, SBP, DBP, WHR, BMI, FBG, PPG, TG, UA, ALT, AST and the prevalence rates of impaired glucose regulation (IFG, IGT, IFG/IGT, DM) increased significantly after adjustment for age and gender (all *P*<0.05). And the Mets and its individual components (including hypertension, dyslipidaemia) also increased across the LFC quintiles (all *P*<0.001). On the other hand, HDL-c decreased across the LFC quintiles (*P*<0.001).

**Table 1:** Characteristics of the study participants stratified by the liver fat content.

LFC (%)	<5%	5%∼10%	10%∼15%	15%∼20%	>20%	*P-*value adjusted i
	n = 1048	n = 816	n = 347	n = 352	n = 273	
Demography						
Age (ys)	63.66±9.84	62.10±9.92	61.71±9.02	61.55±8.98	62.28±9.49	
Male (n)	37.9% (397)	33.5% (273)	35.7% (124)	34.4% (121)	37.7% (103)	
Current smoker	14.3% (150)	16.3% (133)	14.4% (50)	18.5% (65)	15.4% (42)	0.379
SBP (mmHg)	133.47±19.20	132.61±19.19	133.53±19.00	137.97±18.04^b,d,^ e	138.78±16.83^b,d,^ e	<0.001
DBP (mmHg)	75.41±10.07	75.63±10.28	76.56±10.16	80.12±10.35^b,d,f^	79.06±10.63^b,d,f^	<0.001
Fat-related						
WHR	0.88±0.07	0.89±0.07^a^	0.91±0.07^b,d^	0.92±0.07^ b,d,f^	0.93±0.06^b,d,f^	<0.001
BMI	23.12±2.95	23.29±2.93	25.11±3.50^b,d^	26.23±3.13^b,d,^ e	26.34±2.93^b,d,^ e,g	<0.001
Glucose-related						
FBG (mmol/L)	5.18±0.88	5.25±0.84^a^	5.38±0.97^b,^ c	5.63±1.19^b,d,f^	5.70±1.51^b,d,f^	<0.001
PPG (mmol/L)	7.06±3.04	7.07±2.87	7.57±2.62^b,d^	8.87±3.49^b,d,f^	9.23±4.21^b,d,f,h^	<0.001
Lipid-related						
TC (mmol/L)	5.06±0.84	5.12±0.90	5.09±0.86	5.24±1.04a,c,e	5.17±0.96	0.026
HDL-c (mmol/L)	1.49±0.38	1.47±0.38	1.37±0.33^b,d^	1.30±0.29^b,d,^ e	1.26±0.29^b,d,f^	<0.001
LDL-c (mmol/L)	2.93±0.77	2.95±0.77	2.94±0.73	3.00±0.85	2.95±0.83	0.65
TG (mmol/L)	1.27(0.94–1.73)	1.33(0.98–1.83)	1.49(1.09–2.01)^b,^ c	1.81(1.30–2.48)^b,d,f^	1.85(1.37.2.60)^b,d,f^	<0.001
Live enzyme-related						
ALT (U/L)^ j^	14 (11–19)	15 (11–19)	17 (13–23)^b,d^	20 (14–29)^b,d,f^	20 (15–27)^b,d,f^	<0.001
AST (U/L)^ j^	20 (17–23)	20 (17–23)	21 (18–24)a,c	20 (17–24)^b,d^	21 (18–25)^b,d,^ e	<0.001
UA (mmol/L)	300.41±72.59	298.74±75.73	320.12±79.03^ b,d^	333.03±80.78^ b,d,^ e	341.17±76.45^ b,d,f^	<0.001
Glucose profiles						<0.001
IFG	7.9% (83)	9.3% (76)	11.5% (40)	10.2% (76)	9.5% (26)	
IGT	11.7% (123)	11.6% (95)	17.9% (62)	21.3% (75)	21.6% (59)	
IFG+IGT	5.0% (52)	5.5% (45)	9.8% (34)	11.1% (39)	9.2% (25)	
New-DM	9.5% (100)	10.2% (83)	12.7% (44)	21.3% (75)	23.1% (63)	
Syndrome-related						
Dyslipidaemia	39.9% (418)	42.2% (344)	50.0% (177)	69.2% (243)	65.2% (178)	<0.001
Hypertension	33.8% (354)	34.7% (283)	39.8% (138)	46.3% (163)	49.8% (136)	<0.001
Metabolic syndrome	26.3% (276)	28.8% (235)	44.4% (154)	59.7% (210)	63.0% (172)	<0.001

Data are means ± SE or percentages or percentages or median (25th to 75th percentiles).

a P<0.05, ^b^P<0.01 vs.quintile 1;

c P<0.05, ^d^P<0.01 vs. quintile 2;

e P<0.05, ^f^P<0.01 vs. quintile 3;

g P<0.05, ^h^P<0.01 vs. quintile 4;

i : adjustment for age and gender;

j: logarithmically transformed when compared.

Abbreviations: SBP: systolic blood pressure; DBP: diastolic blood pressure; WHR: waist-to-hip ratio; BMI: body mass index; FBG: fasting blood glucose; PPG: postload plasma glucose; TC: total cholesterol; HDL-c: high-density lipoprotein cholesterol; LDL-c: low-density lipoprotein cholesterol; TG: triglycerides; ALT: alanine aminotransferase; AST: aspartate aminotransferase; UA: uric acid; IFG: impaired fasting glucose; IGT: impaired glucose tolerance; DM: diabetes mellitus.

### LFC among the Glucose Categories (Fig. 1)

Among the glucose categories, there was a continuous increase in LFC after adjustment for age and gender (NGT: 7.7±0.3%, IFG: 10.0±0.8%, IGT: 11.8±0.5%, IFG+IGT: 11.7±0.9%, new- DM: 12.4±0.6%, P<0.001). The individuals with newly diagnosed DM had more LFC than the others.

### Relationship between Glucose Metabolism and LFC

In univariate logistic regression analysis (Table 2), the higher the LFC levels ascended by each 1%, the greater the risk of prediabetes and diabetes would be (OR 1.052, 95% CI 1.040–1.064, *P*<0.001; OR 1.062, 95% CI 1.048–1.076; respectively, *P*<0.001). The significant relationship between the LFC and prediabetes and DM remained after adjustment for traditional risk factors.

**Table 2:** Association between the liver fat content and prediabetes and diabetes.

	OR^a^	95% CI^a^	P^a^	OR^b^	95% CI^b^	P^b^
Unadjusted association (every 1% LFC increment)	1.052	1.040–1.064	<0.001	1.062	1.048–1.076	<0.001
Multiple adjusted^c^	1.032	1.019–1.045	<0.001	1.017	1.001–1.033	0.040

a : the risk for prediabetes;

b : the risk for new-diabetes mellitus;

c : adjusted for age, gender, alanine aminotransferase, aspartate aminotransferase, uric acid, body mass index, waist-to-hip ratio, systolic blood pressure, diastolic blood pressure, high-density lipoprotein cholesterol and triglycerides.

To further analyze the role of the LFC in determining impaired glucose regulation, we used multiple linear regression analyses. The LFC was significantly associated with the FBG and PPG suggesting that for each 10 percentage increase in the LFC, there was an increase of 0.084 mmol/l and 0.441 mmol/l of FBG and PPG respectively (*P* = 0.001 and *P*<0.01, respectively) (data not shown).

Moreover, we analyzed the association between the LFC categories and the impaired glucose regulation with logistic regressions in three different models (Table 3). LFC quintiles were associated with impaired glucose regulation in adjusted models. The participants with LFC higher than 10% had higher odds ratios of impaired glucose regulation as compared with those with LFC below 10% after adjustment for all confounding risk factors.

**Table 3:** Odds ratios of impaired glucose profiles according to LFC quintiles.

	Model 1		Model 2		Model 3	
	OR (95% CI)	P value	OR (95% CI)	P value	OR (95% CI)	P value
LFC quintiles(%)						
<5 (reference)	1.00		1.00		1.00	
5∼10	1.12(1.92–1.35)	0.266	1.20(0.98–1.46)	0.073	1.13(0.92–1.39)	0.24
10∼15	2.08(1.62–2.66)	<0.001	2.33(1.81–2.99)	<0.001	1.91(1.46–2.49)	<0.001
15∼20	3.42(2.65–4.39)	<0.001	3.94(3.04–5.11)	<0.001	2.43(1.84–3.22)	<0.001
≥20	3.33(2.53–4.40)	<0.001	3.70(2.79–4.91)	<0.001	2.22(1.64–3.01)	<0.001
P for trend value		<0.001		<0.001		<0.001

P value were calculated from the logistic regression models. Model 1 is unadjusted; Model 2 is adjusted for age and gender; model 3 is adjusted for age, gender, ALT, AST, UA, BMI, WHR, HDL, TG, SBP and DBP.

## Discussion

To our knowledge, this study has shown for the first time in a large community-based population the following: 1) the LFC was independently associated with prediabetes and DM, and the glucose levels (including FBG and PPG) were proportional to the degree of hepatic steatosis; 2) The participants with LFC higher than 10% had higher odds ratios of impaired glucose regulation as compared with those with LFC below 10% after adjustment for all potential confounders.

The LFC independently associated with prediabetes and DM, and the glucose levels were proportional to the degree of hepatic steatosis. The mechanisms by which LFC associated with prediabetes or T2DM could not be elucidated in the present study. Given that the liver plays a key role in glucose metabolism, it is reasonable to assume that hepatic steatosis may play a role in the development of T2DM. And it is widely accepted that hepatic steatosis is usually associated with insulin resistance and consequently overproduces glucose [20], [21].

The relationship between hepatic steatosis and glucose categories is in agreement with a recent data of Kantartzis K et al. [22], who found liver fat may be a more important determinant of impaired glucose regulation than visceral fat. However, they did not provide quantitative relationship between glucose metabolism and LFC. In this aspect, a very recent study in 118 obese adolescents carried out by Cali AM et al. found severity of fatty liver is associated with prediabetes [7]. However, they did not evaluate the contribution of the LFC to the abnormal glucose metabolism, and failed to find the relationship between the LFC and DM. Another recent study by Jung CH has found that the fatty liver index as an indicator of hepatic steatosis independently predicted incident diabetes [11]. Yet, they did not measure the LFC. In our study, we used semi-quantitative ultrasonography to measure LFC and found that the LFC is associated with prediabetic and DM phenotypes. Moreover, we found that the participants with LFC higher than 10% had higher odds ratios of impaired glucose regulation as compared with those with LFC below 10% after adjustment for all potential confounders. These findings were supported by previous study that the early phase of beta-cell function was deteriorated as the LFC accumulated to 10% [23]. Therefore, the identification of those patients with more than 10% liver fat content, who should undergo more intensive lifestyle counselling, may improve the prognosis of NAFLD. Prospective studies are still also needed to validate this finding and determine whether people with more than 10% LFC are prone to the development and progression of prediabetes and diabetes.

What do our present findings tell the clinician? First, screening with semi-quantitative ultrasonography may become a useful tool for the early detection of fatty liver and enable clinicians to provide early interventions. Second, we found that an even slightly elevated LFC is associated with an increased glucose dysregulation. This result highlights the early evaluation of hepatic steatosis may be advantageous for the early detection of impaired glucose regulation and improve the prognosis of NAFLD, because DM is a very important prognostic factor indicating the presence of more advanced fibrosis [24].

The present study has several limitations. First, because our study was cross-sectional, the causative nature of the associations cannot be established. Second, hepatic steatosis was based on ultrasound imaging but was not confirmed by liver biopsy. Thus we could not evaluate the relationship between glucose regulation and specific histological features of NAFLD (such as inflammation and fibrosis), which were found to be associated with the presence and severity of the metabolic syndrome [25], [26], [27]. However, previous studies have found that the more the fat accumulated in the liver, the more likely the presence of histological findings would be compatible with NASH [28], [29]. Therefore, the results can also reflect the relationship between NASH and glucose regulation in a certain degree.

In conclusion, we found that the LFC is associated with prediabetic and DM phenotypes and thus may be considered a strong risk factor for T2DM in middle-age and elderly, independent of confounding risk factors. The risks for prediabetes and diabetes are proportional to the degree of hepatic steatosis. And even a slightly elevated LFC is associated with abnormal glucose metabolism. Future prospective studies are necessary to validate these findings and better determine the cut-off value for risk of adverse glucose metabolism.
